# Neck muscle responses of driver and front seat passenger during frontal-oblique collisions

**DOI:** 10.1371/journal.pone.0209753

**Published:** 2018-12-31

**Authors:** Andreas Mühlbeier, Kim Joris Boström, Wolfram Kalthoff, Marc H. E. de Lussanet, Cassandra Kraaijenbrink, Lena Hagenfeld, William H. M. Castro, Heiko Wagner

**Affiliations:** 1 Department of Movement Science, University of Münster, Münster, Germany; 2 Otto Creutzfeld Center (OCC), University of Münster, Münster, Germany; 3 OFI Orthopädisches Forschungsinstitut, Münster, Germany; University of Illinois at Urbana-Champaign, UNITED STATES

## Abstract

**Background:**

Low-velocity motor vehicle crashes often lead to severe and chronic neck disorders also referred to as whiplash-associated disorders (WAD). The etiology of WAD is still not fully understood. Many studies using a real or simulated collision scenario have focused on rear-end collisions, whereas the kinematics and muscular responses during frontal-oblique collisions have hardly been investigated. In particular for rear-end collisions, drivers were shown to have a higher WAD risk than front seat passengers. Yet, independently from the impact direction, neither the muscular nor the kinematic responses of drivers and front seat passengers have been compared to date, although some findings indicate that the neck muscles have the potential to alter the head and neck kinematics, and that the level of neck muscle activity during impact may be relevant for the emergence of WAD.

**Objective:**

In this study, we quantitatively examined the subjects’ neck muscle activity during low-velocity left-frontal-oblique impacts to gain further insights into the neuromuscular mechanism underlying whiplash-like perturbations that may lead to WAD.

**Methods:**

In a within-subject study design, we varied several impact parameters to investigate their effect on neck muscle response amplitude and delay. Fifty-two subjects experienced at least ten collisions while controlling for the following parameters: change in velocity Δ*v* (3 / 6 km/h), seating position (driver / front seat passenger), and deliberate pre-tension of the musculature (tense / relaxed) to account for a potential difference between an expected and an unexpected crash. Ten of the 52 subjects additionally ran the same experimental conditions as above, but without wearing a safety belt.

**Findings:**

There were significant main effects of Δ*v* and muscle pre-tension on the reflex amplitude but not of seating position. As for the reflex delay, there was a significant main effect of muscle pre-tension, but neither of Δ*v* nor of seating position. Moreover, neither the safety belt nor its asymmetrical orientation had an influence on the reflexive responses of the occupants.

**Conclusion:**

In summary, we did not find any significant differences in the reflex amplitude and delay of the neck musculature between drivers and front seat passengers. We therefore concluded that an increased risk of the driver sustaining WAD in frontal-oblique collisions, if it exists, cannot be due to differences in the reflexive responses.

## Introduction

In 2016, there were around 307.500 physical injuries sustained from motor vehicle crashes in Germany [1]. Almost half of them suffered from whiplash-associated disorders (WAD) [2, 3]. The etiology of WAD is not fully understood. Initially, a cervical hyperextension was thought to be the responsible mechanism [4, 5]. Later studies discussed the role of high pressure in the spinal canal [6], abnormal loading of the vertebrae [7, 8], overstretching of muscles and ligaments [9], and hypertranslation of individual vertebrae [10–12]. These physical explanations all seek the underlying injury mechanism in the relative motion of the head to the torso [13]. In addition to such physical factors, there is evidence that psychological factors may play an important role, too [14]. The etiology of WAD may further depend on various occupant and crash-related factors such as the change in velocity of the vehicle (Δ*v*) [15], the age, constitution and gender of the passenger, their seating position, and the impact direction [16].

Frontal-oblique collisions typically occur during intersection accidents and sliding collisions and are much less investigated than rear-end or frontal collisions. An analysis of 1001 individual medical examinations in the context of litigations and insurance claims concerning WAD showed that 56% had undergone a rear-end collision, while 16.7% had undergone a frontal-oblique, 7.7% lateral, 7.4% frontal, 1.6% rear-oblique, and 10.6% another kind of collision (collection period 2011–2014 in Germany; W.H.M. Castro, non-published data). Most of the available data on frontal-oblique collisions stem from investigations using post mortem human subjects [17] or crash test dummies [18], and could therefore not consider neuromuscular behavior. The above numbers illustrate the requirement of investigations with a frontal-oblique collision direction and human living subjects.

In rear-end collisions, occupants in the front seats were shown to have a higher risk of sustaining WAD than those in the rear seats, and drivers had a higher risk than front seat passengers (FSPs) [19, 20]. The increased risk for drivers with respect to FSPs was also found when all kinds of collisions regardless the impact direction were included [16]. However, to date there is no study comparing the driver’s and FSP’s neck muscle activity, neither in rear-end nor in any other impact directions.

The onset of muscle reflexes were once believed to be too late to influence the head’s kinematics during a crash [21–23]. More recently, however, it was suggested that for low velocity crashes the neck muscles are active during the interval in which the WAD injury is likely to occur [9, 12, 24]. Thus, the neck muscle activity might indeed influence the emergence of WAD. It is not known if the neck muscle activity increases the risk and severity of WAD, or whether it has a protective effect and thus reduces the risk of it [25].

Brault et al. (2000) [9] exposed 42 subjects to rear-end collisions of 4 km/h and 8 km/h speed change, and identified that a Δ*v* of 8 km/h induced significantly higher reflex amplitudes and earlier muscle activation times in sternocleidomastoid (SCM) and paraspinal (PARA) muscles than a Δ*v* of 4 km/h.

The cervical muscle response during a collision might be triggered by various stimuli, such as the loud noise, the impact-induced vehicle motion, vibration, and whole-body motion [26]. The vestibular (vestibulocollic) reflex [12, 27–29], the startle reflex [12, 25, 30–32], postural responses mediated by more distal mechanoreceptors [12], as well as stretch reflexes of neck muscles (cervicocollic reflex) [12, 26, 28] might all play a role in the muscular responses.

Furthermore, collision awareness may result in a significantly lower frequency of several symptoms and a lower intensity of headache pain [33]. Unpreparedness, on the other hand, was associated with poor recovery 12 weeks after injury [34]. A pre-tensioning of the cervical muscles has been shown to reduce the motion of the head relative to the neck, in terms of distance and acceleration [22, 35] by 30% [36]. Accordingly, it has been suggested that the awareness of an impending impact might reduce the risk and severity of WAD [35, 36]. The reflex onset times of cervical muscles appear to be independent of awareness and predictability of an upcoming collision [37, 38].

In order to gain further insights into the neuromuscular mechanism underlying whiplash-like perturbations that may lead to WAD, we systematically measured the influence of the relevant parameters on neck muscle activity using a relatively large population of 52 voluntary subjects to obtain sufficient statistical power. The subjects experienced low-velocity from a left-frontal-oblique direction. In a full within-subject study design, we measured neck muscle response amplitude and delay. The independent variables were impact velocity change Δ*v* (3 / 6 km/h), seating position (driver / front seat passenger), deliberate pre-tension of the musculature (tense / relaxed). Each subject experienced at least ten collisions, including two familiarization trials.

There is no doubt that safety belts play a crucial role in preventing injuries during accidents at high velocities. It is less obvious, however, whether such a protective function also applies at lower velocities and, in particular, whether there is any measurable effect of wearing a belt on the neck muscular response. The backrest of the seat will be pushed against the occupant’s back during a rear-end collision. However, during a frontal or frontal-oblique collision, the backrest is pushed away from the occupant, who is pressed into the belt. We, therefore, conducted an additional experiment to measure the influence of wearing a safety belt during frontal-oblique collisions.

Based on the above presented literature we derived and tested the following four hypotheses (H1-H4). As the driver has been reported to suffer from WAD more often than the FSP, and as the cervical muscle activity is likely to influence the head-neck movements and accelerations, we hypothesized that the driver’s and FSP’s cervical muscle responses differ in reflex amplitude (H1a), reflex delay (H1b), and that the relative head-torso movement, velocity, or acceleration differ as well (H1c).

Expectation of an incoming collision has been shown to not significantly influence the onset of the neck muscles. Since subjects tend to tense their muscles when anticipating a crash, it is plausible to expect a difference in the magnitude of the muscular response. We thus hypothesized that pre-tensioning of the neck muscles will affect the reflex amplitude (H2a), but not the reflex delay (H2b) of the cervical muscular responses.

Thirdly, motivated by the reported influence of Δ*v* on the muscular response, we hypothesized that in frontal-oblique collisions, a Δ*v* of 6 km/h elicits higher reflex amplitudes (H3a) and shorter reflex delays (H3b) of the cervical muscular responses than a Δ*v* of 3 km/h does.

Finally, with respect to the influence of wearing a 3-point lap and shoulder belt during frontal-oblique collisions, we hypothesized that wearing a belt has an effect on the reflex amplitude (H4a), and the reflex delay (H4b) of the cervical muscular responses.

## Materials and methods

### Subjects

Sixty-five healthy subjects participated in the experiment. The data of 13 of them had to be excluded due to technical problems with either the electromyographic (EMG) data or the kinematic data. Of the remaining 52 subjects, 30 were male and 22 were female (age: 32 ± 14 years, body mass: 72 ± 13 kg, and body height: 178 ± 9 cm). All subjects signed their informed consent form that had been approved by the local ethics committee of the Department of Sports Science and Psychology of the University of Münster. All subjects possessed a driving license and were paid nominal amount for the participation in the study. Besides, none of them was pregnant or suffered from neck or back pain. The individuals depicted in the figures of this manuscript have given written informed consent to publish these case details (as outlined in the PLOS consent form).

### Experimental setup and procedure

#### Setup of the crash vehicle and the towing vehicle

**Fig 1:**
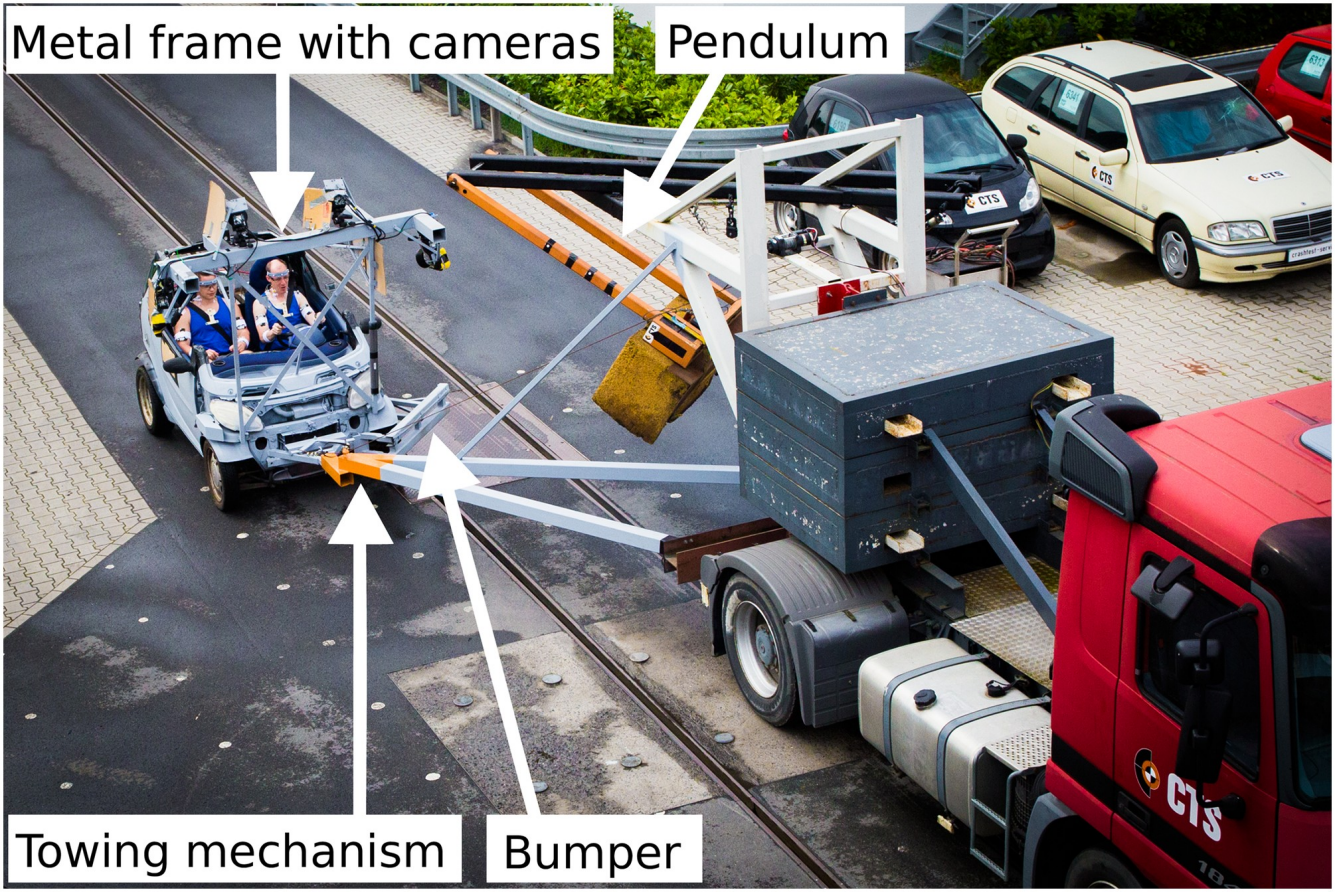
Crash scenario. Scenario of the collision with the towing vehicle drawing the crash vehicle with the help of the towing mechanism. Four motion capture cameras (Qualisys, Sweden) and two video cameras (GoPro, USA) were mounted to a metal frame at the front of the crash vehicle. The pendulum impacted the bumper from a left-oblique direction.

A Smart Fortwo (curb weight: 513 kg; year of manufacture: 2000, Fig 1) was chosen as the crash vehicle by virtue of its low weight. This model has two seats, one for the driver and one for the FSP. The mass of the crash vehicle was further reduced by removing the engine and the gearbox. Moreover, the A-pillars, the windshield, and parts of the steering wheel were removed to improve the motion capture. To enable motion capture of the participants, a stiff metal construction was added to the front of the crash vehicle (Fig 1). The crash vehicle was drawn by a truck with the help of a towing mechanism that was fixed laterally to the truck (the mounting point is orange in Fig 1). A metal bumper was added at the site of impact to protect the wheel housing. A tape switch on the surface of the pendulum buffer produced a flash light on each impact. A 12 volt truck battery provided power for the motion capture cameras and the EMG device.

The truck was prepared with a pendulum to impact the bumper of the crash vehicle. A foam block was used to soften the impact of the pendulum and to generate a realistic impact duration of approximately 0.1 s. The truck driver controlled a trigger mechanism that simultaneously released the crash vehicle and the pendulum.

#### Procedure and protocol

The subjects were measured in pairs. After being prepared with EMG electrodes and retro-reflective markers, the subjects adjusted their seats individually and connected their seat belts. One of the experimenters read out the experimental condition and the instructions. The driver was instructed to steer straight while the car was drawn by the truck. The pendulum was either dropped from a low or high position to cause a velocity change (Δ*v*) of either 3 or 6 km/h. An incident data recorder measured the acceleration of the crash vehicle during the impact to control for the proper change in velocity Δ*v*. In the ‘tense’ condition the instruction (translated from German) was:

‘You are driving in a car and see another car approaching you from the left side, which has overlooked you. A collision is unavoidable. Consequently, you adopt a tense posture briefly before the crash.’

In the ‘relaxed’ condition the instruction was as follows:

‘You are driving in a car. Suddenly a collision hits you from the left side. Since the collision hits you unexpectedly, your posture is relaxed.’

The drivers were instructed to keep their hands on the steering wheel, whilst the FSP put their hands in their lap.

Before each run, the vehicle was mounted to the truck and the pendulum lifted to its initial position. The EMG recordings were started first, followed by the interior and exterior motion capture system that began their measurement synchronously on pressing the shared trigger button. The truck accelerated the crash vehicle to a speed of approximately 30 km/h. After reaching a location marked on the track, the truck driver pressed the release button to release both the crash vehicle and the pendulum. The pendulum impacted the bumper on the frontal-oblique direction side of the crash vehicle causing a resulting change in velocity of 3 or 6 km/h and a slight change of the driving direction to the right. As soon as the vehicle had assumed a straight and stable course, the driver stopped the vehicle.

At least ten runs were recorded for each pair of participants (Table 1). The first two runs were familiarization trials in tense posture with a Δ*v* of 3 km/h (first) and 6 km/h (second).

**Table 1:** Test protocol of the main experiment. (*N* = 42).

Run No.	Driver	FSP	Δ*v* (km/h)	Muscle tone
1	subject A	subject B	3	tense
2	subject A	subject B	6	tense
3-6	subject A	subject B	3 or 6	tense or relaxed
7-10	subject B	subject A	3 or 6	tense or relaxed

After the familiarization runs, four experimental conditions with two different instructions and two different velocity changes were recorded in randomized order. After that the occupants changed the seating position so that the driver became the FSP and vice versa. Again, the four experimental conditions were carried out in a randomized order. In case of a technical failure the run was repeated at the end of the four experimental runs. Since data processing was largely performed at a later time, a run was also repeated if the experimenters only suspected a technical failure (e.g. an impact outside the recording range of the external motion capture system).

All 52 subjects experienced 8 experimental conditions from all combinations of three independent variables: seating position (driver / FSP), muscle tension (tensed / relaxed), and change in velocity Δ*v* (3 km/h / 6 km/h).

#### Additional experiment: Belt vs. no belt

An additional experiment was carried out to test the influence of the 3-point lap and shoulder belt on the muscular and kinematic responses of the occupants. Ten of the subjects experienced eight runs in addition to those of the main experiment, but this time without wearing a belt. To limit the number of runs per subject, the familiarization trials were left out for these ten subjects. Thus, the total number of runs for these subjects was at least 16 (Table 2). All runs were randomized with evenly distributed attributes for every independent variable. The remaining procedure stayed the same.

**Table 2:** Test protocol of the belt experiment. (*N* = 10).

Run No.	Driver	FSP	Δ*v* (km/h)	Muscle tone	Belt
1-4	subject A	subject B	3 or 6	tense or relaxed	belted
5-8	subject A	subject B	3 or 6	tense or relaxed	unbelted
9-12	subject B	subject A	3 or 6	tense or relaxed	belted
13-16	subject B	subject A	3 or 6	tense or relaxed	unbelted

### Electromyography

**Fig 2:**
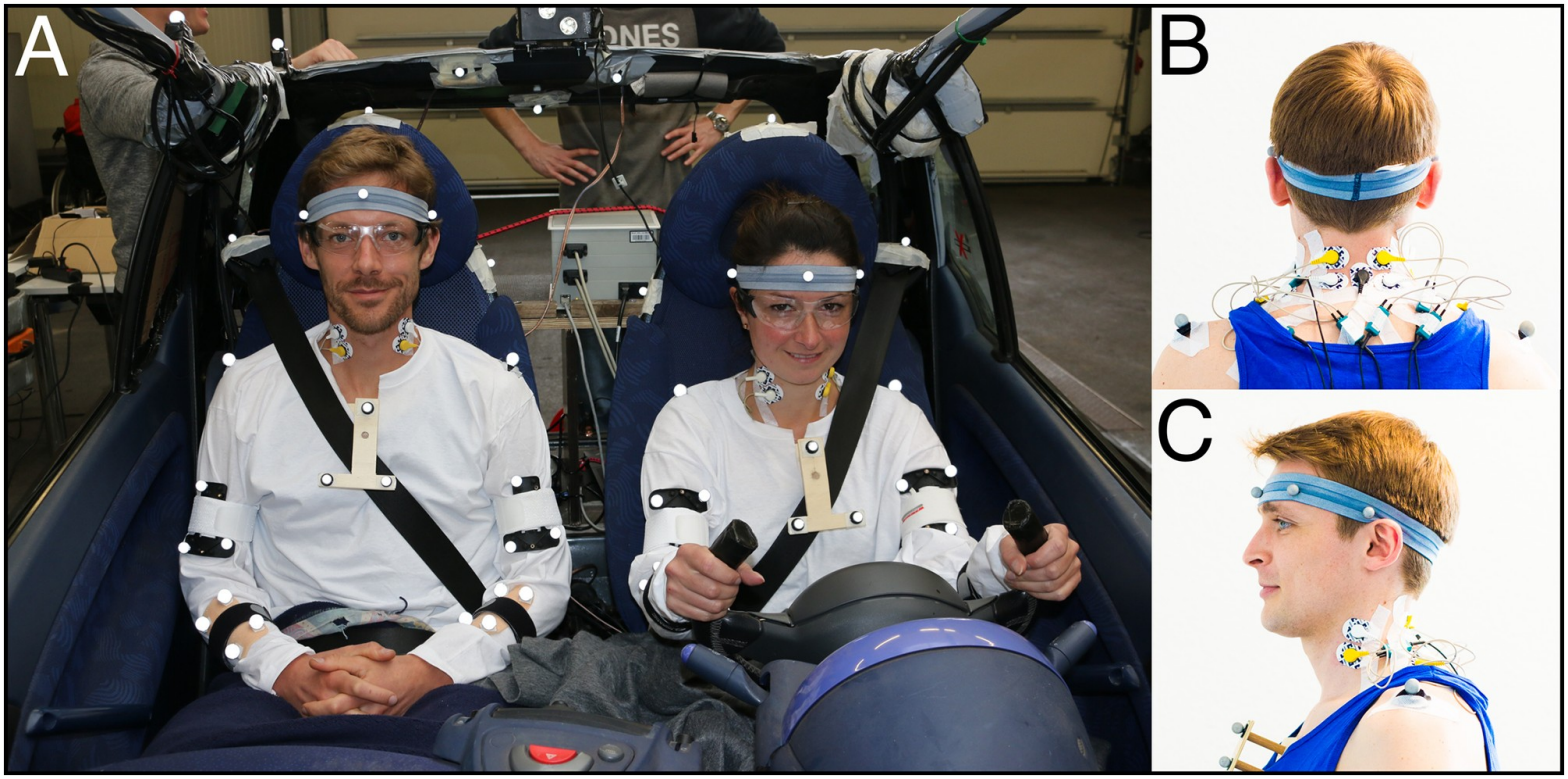
Subjects. (A) Anterior view of subjects sitting in driver and FSP position. Infrared-reflective markers were attached at anatomical landmarks. The LED-flash (at the middle-top of the figure) was triggered when the pendulum hit the bumper. The steering wheel was trimmed to provide a better view for the motion capture cameras. (B) Posterior (C) and lateral view of the subjects. The EMG electrodes were placed at the sternocleidomastoid, trapezius, and paraspinal muscles.

Surface electromyograms of the sternocleidomastoid (SCM), trapezius (TRA), and cervical paraspinal (PARA) muscles were bilaterally recorded with two different EMG systems (DeMeTec, GJB Datentechnik GmbH, Germany and Biovision, Germany; preamplified 1.000 times). As the posterior neck muscles are arranged in multiple layers, the term *paraspinal muscles* (PARA) actually represents the total muscle activity recorded at these electrodes [31]. After the skin had been shaved and cleaned with medical abrasive paste (OneStep, H+H Medizinprodukte GbR, Münster, Germany), disposable Ag/Ag-Cl electrodes (H93SG, Covidien, Neustadt, Germany) with a circular uptake area of 0.5 cm in diameter were placed with an inter-electrode distance of 2.5 cm. The electrode placement was done according to Hermens et al. (1999) [39] and Falla et al. (2002) [40]. For the SCM, the electrodes were attached along the sternal portion of the muscle, with the electrode center at 1/3 of the distance between the sternal notch and the mastoid process [40] (Fig 2C). For the TRA, the electrodes were positioned halfway between the 7th cervical vertebra (C7) and acromion while the PARA-electrodes were located laterally to the spinal column directly under the hairline (Fig 2B). The common ground electrode was affixed at C7. Tape was used to secure the electrodes. Muscle activity was measured bipolar at a sampling rate of 2000Hz (ToM Erfassung, GJB Datentechnik GmbH, Germany).

### Kinematics

Retro-reflective markers were attached to the head, breast, shoulders, upper arms and forearms (Fig 2A). Except for the shoulder markers, all subject markers were grouped in clusters of three or four so that the 3D orientation of the body segment could be computed in addition to the 3D position. Additionally, markers were placed inside and outside the crash vehicle as well as on the seats. The four high-speed motion capture cameras (Oqus 300, Qualisys, Sweden) that were mounted at the front side of the car recorded the 3D movements of retro-reflective markers at a 250 frames per second (fps). The distance from the starting point to the location of the crash was approximately 85 meters. At the crash location a series of ten further motion capture cameras (Oqus 400, Qualisys, Sweden, 250 fps) recorded the 3D kinematics of the vehicle around the time of impact, for about 14 m along the driving direction. Three markers were recorded by both the interior and exterior motion capture system to align the coordinates of the interior and the exterior motion capture recordings. A shared trigger construction ensured that the capture of the interior and the exterior motion capture systems were synchronized. Using this combined motion capture recording allowed us to compute the earth-bound motions of the participants.

### Data analysis

The processing of the kinematic and EMG data was performed using MatLab (version 2017b, MathWorks, USA).

#### EMG data

Initially, the activity of the tape switch caused an artifact in some of the electrodes. In most cases this artifact had the shape of a sharp block pulse which could be easily subtracted from the data. In cases where this was not possible the affected EMG signal was excluded from the statistical analysis.

The 12V battery current was transformed to approximately 50Hz 220V by an on-board current transformer. Unfortunately, this sometimes caused electromagnetic noise of approximately 50 Hz plus harmonics that contaminated the EMG signals. The EMG signals were filtered by a periodic moving average that was subtracted from each signal individually. A Fast Fourier Transformation (FFT) on the thus cleaned data showed a resulting smooth spectrum in the noise frequencies, even for the most severely perturbed signals.

Consecutively, all EMG signals were high-pass filtered (4th-order Butterworth filter, 20 Hz [41]), rectified, and smoothed by a ± 10 samples moving average. The reflex onset was defined at the first sample where the signal exceeded 5 standard deviations above baseline activity, which in turn was calculated as the mean over a period of 300 ms before impact. Instead of simply taking the maximum value, the reflex amplitude was determined as follows. First, all extreme values (local maxima and minima) within 20 to 300 ms after response onset were collected. Then, if a local maximum was followed by a local minimum within 10 ms, the maximum was dismissed. From the remaining local maxima, the first one was taken as the reflex amplitude. Thus, if there were two separate reflex components, the first one was taken into account even though the second might have had a higher amplitude.

#### Reconstruction of the impact time

As the tape switch signal introduced artifacts in the EMG data, we disabled the tape switch and determined the impact time by analyzing the kinematics of the stationary markers in the passenger compartment of the vehicle as follows. The pendulum’s impact slightly deformed the vehicle which caused the passenger compartment markers to move in a highly specific manner. This was exploited to reliably determine the impact time. As the cameras were mounted on a solid construction, the 3D image itself did not suffer. Two raters independently from each other always selected the same frame as the impact time, i.e. 4 ms accuracy at 250 Hz measurement rate.

#### Kinematic data

The 3D positions and orientations of markers and rigid bodies (calculated using the clusters) were computed using the standard Qualisys analysis software (QTM version 2.16) and exported to Matlab files. The coordinate system was then aligned to the pre-impact driving direction, from the recorded vehicle movement in the external motion capture system. The internal coordinate system was aligned to the external one using a Procrustes analysis on the passenger compartment markers that were recorded by both systems at the time of impact.

To correct for the deformations of the vehicle, the average motion of the gaps of up to 100 ms were filled using the Matlab polyfit function, with a 4th order polynomial and a fit window with tails of 13 samples (56 ms).

#### Statistical analysis

Due to measurement-related difficulties, not all of the collected data was usable. This led to an unbalanced data structure where not all experimental conditions yielded the same number of data points. Consequently, a standard N-way repeated measures ANOVA was not an option, so instead we performed either a linear mixed model (LMM) analysis, or, for those cases where the response variable was strictly positive (reflex amplitude and delay), a generalized linear mixed model (GLMM) analysis. The LMM and GLMM models were fitted with the maximum-likelihood method using the statistical software R with the package ‘lmerTest’ and the functions lmer for LMM and glmer for GLMM. To obtain p-values for the main effects and interaction effects, a type-3 ANOVA with *χ*^2^-statistics was performed on each fitted model using the command Anova from the ‘car’ package. The package ‘emmeans’ and its functions emmeans and pairs was used to perform Holm-Bonferroni corrected post-hoc multiple-comparison tests.

For the GLMMs, we chose a logarithmic link function and gamma distributed errors. This choice is the most generic one for continuous and strictly positive response variables, and, what’s more, none of the other possible choices yielded convergence. The LMMs were performed with an identity link function and a Gaussian error distribution. We chose to perform the more conservative type-3 ANOVA in view of the potential and actual interaction effects, and we based it on *χ*^2^-tests rather than F-tests, because this type of test is more robust against a non-normal distribution of data. The appropriate effect size parameter for *χ*^2^-tests is not $\eta_{p}^{2}$ but Cramer’s *ϕ* (also known as Cramer’s *V*), which we calculated for each significant effect. The given interpretation of each effect size as *small*, *medium*, or *large*, depends on the degree of freedom (*df*) and was interpreted according to Cohen’s rule of thumb [42] in the following way: For *df* = 1, a small/medium/large effect corresponds to values of *ϕ* around 0.1/0.3/0.5, and for *df* = 2, to values around 0.07/0.21/0.35. Since centering and scaling does not effect statistical inference in regression models, no data normalization was carried out.

We considered the following continuous dependent variables of interest: the reflex amplitude, the reflex delay, the path length of the movement of the head in relation to the torso, the velocity of this movement, and its acceleration. Each of these variables were modeled to depend on a selection of the following categorical independent variables: change in velocity (DeltaV = 3 or 6 km/h), seating position (Seat = driver or front seat passenger), deliberate pre-tension of the musculature (Tension = tense or relaxed), measured muscle type (Muscle = PARA, SCM, or TRA), and the condition of wearing a safety belt or not (Belt = belted or unbelted). We then considered the unique subject ID (id) as a categorical variable with as many levels as there are subjects, to enter the modeling of random effects. As the EMG data were obtained using two different measurement systems with different output levels, we included an additional variable EMGSys to account for, and thus eliminate, these level differences. Altogether, based on recommendations of Wollschläger [43], the fixed and random effects were modeled according to the schema

$$\begin{array}{c} Y∼A*B*C+\text{EMGSys}+(1|\text{id})+(1|\text{id}:A)+(1|\text{id}:B)+(1|\text{id}:C), \end{array}$$ (1)

where Y stands for the dependent variable under consideration, and A, B, and C are placeholders for a varying number of independent variables under consideration. For the kinematic measurements the term EMGSys was omitted. Note that terms of the form A*B*C are expanded to 1 + A + B + C + A:B + A:C + B:C + A:B:C by the lmer algorithm of R, where the term 1 represents the intercept, single terms—like A, B, C—represent fixed main effects, colon-separated terms—like A:B—represent fixed interaction effects, and bracketed terms—like (1|A)—represent random effects. It is important to acknowledge that in the presence of significant interactions between independent variables, the corresponding main effects may not have a straightforward interpretation and must be handled with caution.

**Fig 3:**
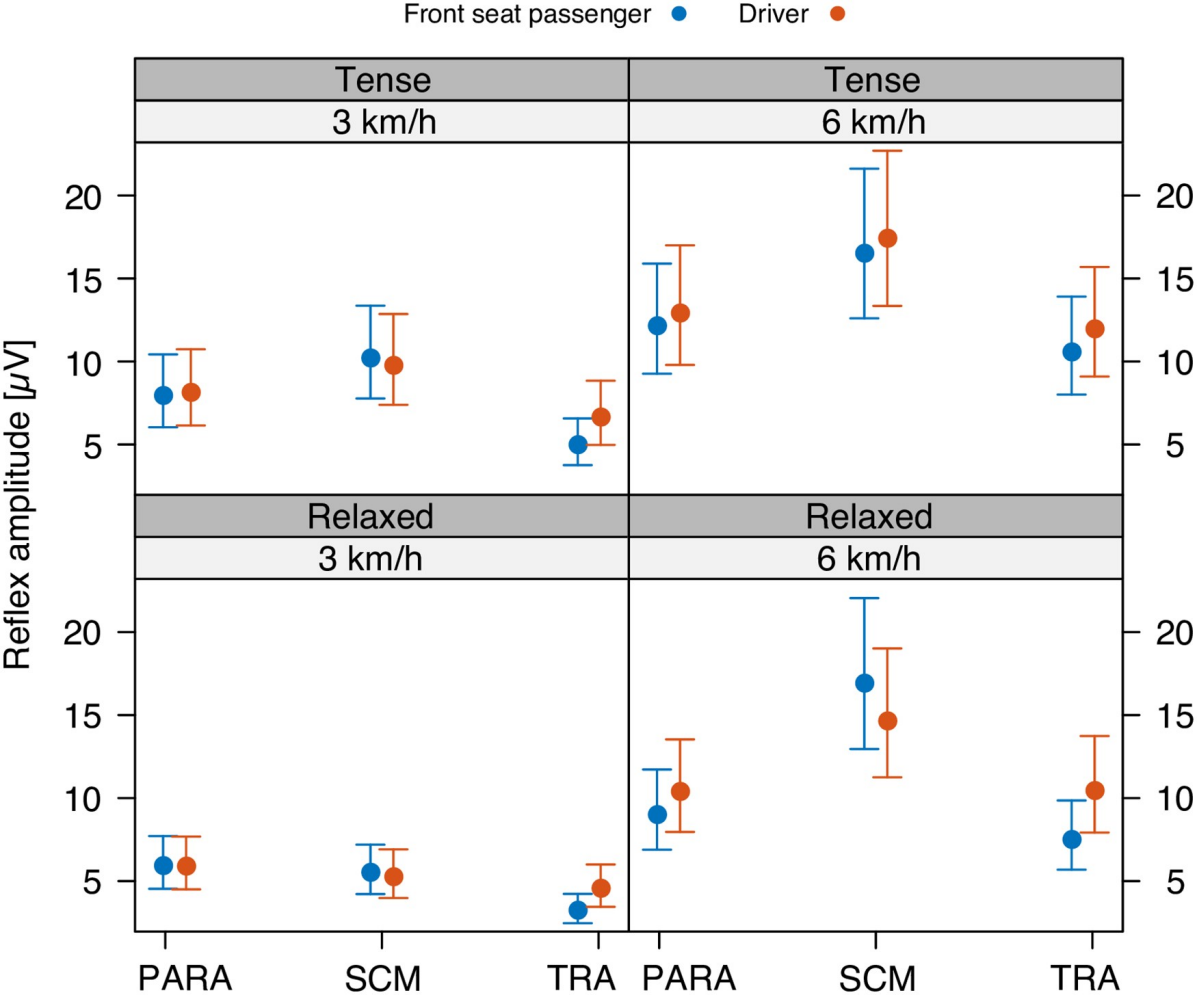
Reflex amplitude. Mean and 95%-confidence interval of the reflex amplitude of cervical paraspinal (PARA), sternocleidomastoid (SCM), and trapezius (TRA) muscles in driver and front seat passenger position following a Δ*v* of 3 (left) and 6 km/h (right), and in relaxed (bottom) and tense condition (top).

**Fig 4:**
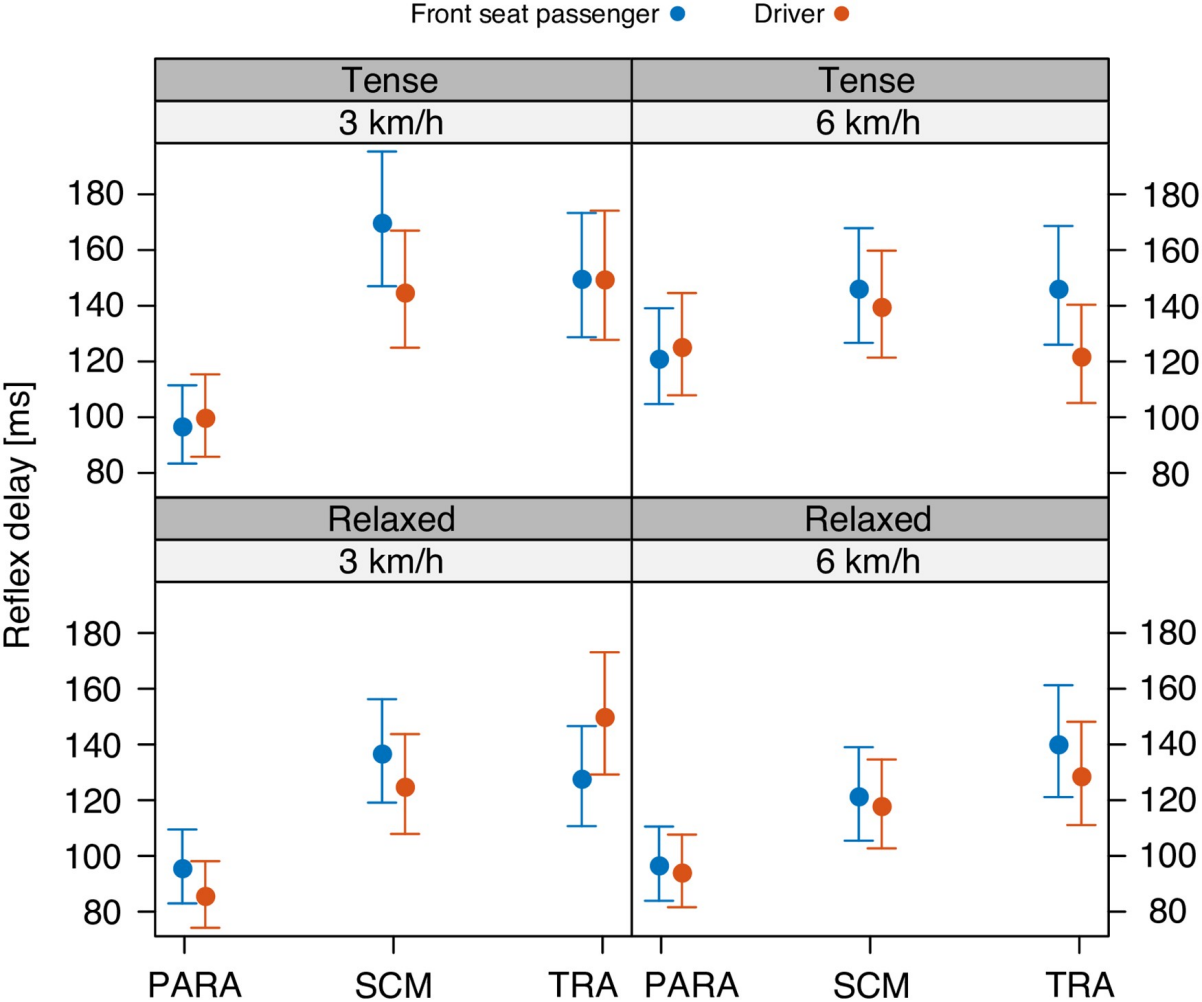
Reflex delay. Mean and 95%-confidence interval of the reflex delay of cervical paraspinal (PARA), sternocleidomastoid (SCM), and trapezius (TRA) muscles in driver and front seat passenger position following a Δ*v* of 3 (left) and 6 km/h (right), and in relaxed (bottom) and tense condition (top).

Our first two GLMM analyses were performed with the dependent variable being either the reflex delay or the reflex amplitude, and with the independent variables being DeltaV, Seat, Tension, and Muscle. These two analyses are associated with, respectively, Figs 3 and 4, and Tables 3 and 4.

**Table 3:** Main and interaction effects calculated by the GLMM analysis of the reflex amplitude. Only significant effects are shown, p-values are rounded up to the next significant digit. Effect sizes are given as Cramer’s *ϕ* with a corresponding interpretation (small, medium, large).

Source	*χ*^2^	Df	*p* <	*ϕ*	
Muscle	29.48	2	0.0001	0.12	medium
Tension	23.69	1	0.0001	0.15	medium
Δ*v*	120.86	1	0.0001	0.34	large
EMG system	9.32	1	0.01	0.09	small
Seat x Muscle	15.53	2	0.001	0.09	medium
Muscle x Δ*v*	20.53	2	0.0001	0.10	medium
Tension x Δ*v*	14.15	1	0.001	0.12	medium
Muscle x Tension x Δ*v*	10.49	2	0.01	0.07	medium

**Table 4:** Main and interaction effects calculated by the GLMM analysis of the reflex delay. Only significant effects are shown, p-values are rounded up to the next significant digit. Effect sizes are given as Cramer’s *ϕ* with a corresponding interpretation (small, medium, large).

Source	*χ*^2^	Df	*p* <	*ϕ*	
Muscle	60.62	2	0.0001	0.17	medium
Tension	9.24	1	0.01	0.09	small
Muscle x Δ*v*	23.44	2	0.0001	0.11	medium
Muscle x Tension	8.54	2	0.05	0.06	small
Seat x Muscle x Δ*v*	8.88	2	0.05	0.06	small

**Fig 5:**
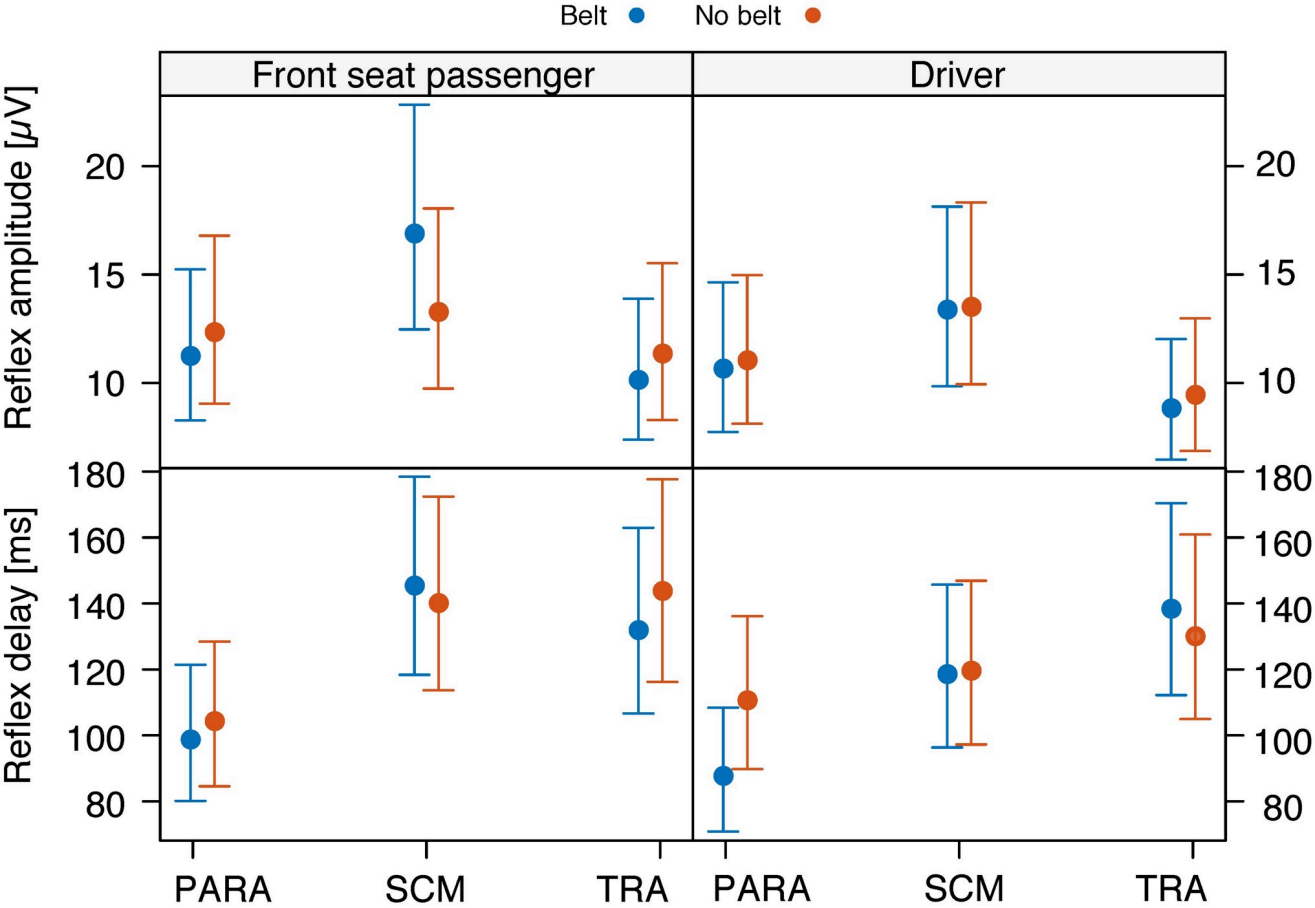
Effect of the safety belt on the EMG. Mean and 95%-confidence interval of the reflex amplitude (top) and the reflex delay (bottom) of the cervical paraspinal (PARA), the sternocleidomastoid (SCM), and the trapezius (TRA) muscles in the belted and not belted condition.

Ten of the 52 subjects additionally ran the same experimental conditions as in the main experiment, but this time without wearing a belt. This made it possible to analyze the effect of the belt as another within-subject factor on a subset of the original subject pool. Hence, the third and fourth GLMM analyses, associated with Fig 5, were performed on this sub-ensemble using schema (1) with the dependent variable being, again, either the reflex delay or the reflex amplitude, and with the independent variables being Seat, Muscle, and Belt. These analyses are associated with Fig 5.

**Fig 6:**
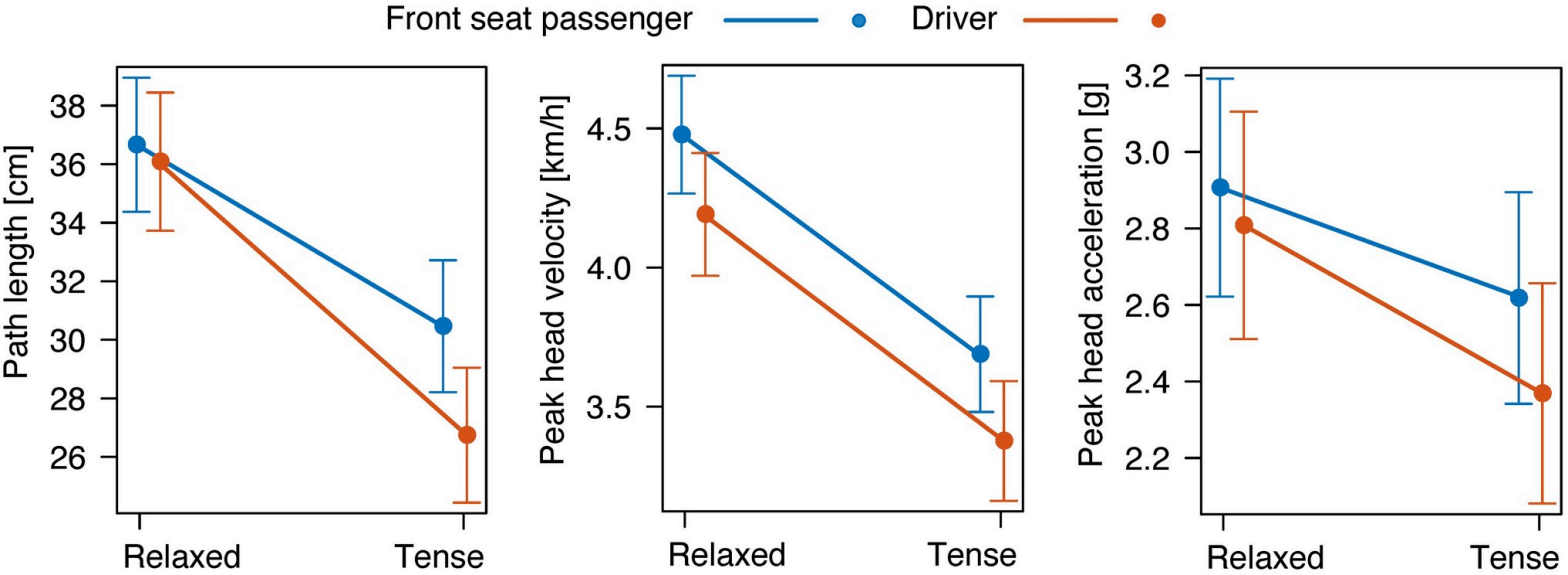
Kinematics. Mean and 95%-confidence interval of the path length of the head in relation to the torso (left), the maximum velocity of the head in relation to the torso (middle), and the maximum acceleration of the head in relation to the torso (right) of 52 subjects in driver and front seat passenger position following collisions with a change in velocity of 6 km/h.

LMM analyses 5, 6, and 7 have been performed with the dependent variable being either the path length, the velocity, or the acceleration of the head in relation to the torso, and with the independent variables being Seat and Tension. The path length of the head movement in relation to the torso was calculated as

$$\begin{array}{cc} L & =\sum_{i=1}^{N-1}∥x_{i+1}-x_{i}∥, \end{array}$$ (2)

where ***x***_*i*_ is the *i*-th sample of the three-dimensional trajectory, *N* is the total number of samples, and ‖ ⋅ ‖ denotes the Euclidean norm. The data have been conditioned on a change in velocity of Δ*v* = 6 km/h, the reason being that 1) the main focus in this part of the analysis is not on the change in velocity but on the influence of the seating position, and 2) the differences of the dependent variable were more pronounced at Δ*v* = 6 km/h. The kinematic comparison of Δ*v* = 3 km/h against Δ*v* = 6 km/h will be carried out in a later study. These analyses are associated with Fig 6.

## Results

With respect to hypothesis H1, the statistical analysis reveals a significant difference in the kinematic response of driver and FSP in terms of the path length and the velocity of the movement of the head relative to the torso. There was, however, no significant difference with respect to the acceleration of the head-torso movement, nor with respect to the muscular response amplitude or delay. Hence, hypotheses H1a and H1b must be rejected, while H1c is supported by the data.

As for hypothesis H2, the pre-tensioning of the musculature had a significant effect on both the reflex amplitude and delay. More precisely, the reflex amplitude increases and the delay decreases when the musculature is pre-tensed. Hence, hypothesis H2a is supported by the data, while H2b is not.

Regarding hypothesis H3, the higher Δ*v* caused a significantly higher reflex amplitude in the neck musculature, while there was no significant main effect of Δ*v* on reflex delay. There was, however, a significant interaction between Δ*v* and muscle type, as well as between Δ*v*, muscle type, and seating position. This means that the corresponding calculated main effects have no straightforward interpretation, since the interaction indicates that the muscle delay of some muscles changed differently when Δ*v* was increased, and when the seating position was changed. In summary, hypothesis H3a is supported by the data, while, in the sense of a direct impact of Δ*v* on response delay, hypothesis H3b remains unsupported.

The safety belt neither had a significant influence on the reflex amplitude nor on the delay (*p* > 0.98 and *p* > 0.72, respectively). Hence, both of our hypotheses H4a and H4b are not supported by the data.

In the following we give a detailed report of the statistical results.

### Reflex amplitude

As for the reflex amplitude (Fig 3), the GLMM analysis (Table 3) revealed a significant interaction between the three variables muscle type, tension, and Δ*v* (*p* < 0.01). Also, there turned out to be significant interaction effects between muscle type and Δ*v* (*p* < 0.0001), and between muscle type and seating position (*p* < 0.001). These interaction effects entail that the corresponding main effects must be treated with caution, as calculating a main effect amounts to averaging over the corresponding conditional effects. A significant main effect implies that there is an overall increase or decrease of the observable under consideration (here, reflex amplitude) depending on the respective variable.

There was no significant main effect (*p* > 0.15) of the seating position on the reflex amplitude. There was, however, a significant main effect of muscle tension on the reflex amplitude (*p* < 0.0001). Taking a tense body posture prior to the impact trended towards a greater reflex amplitude in comparison to an initially relaxed body posture, especially in the Δ*v* = 3 km/h condition (Fig 3, top vs. bottom). The post-hoc tests showed that this trend only reached significance in the SCM muscles of both the FSP and the driver (Fig 3, compare relaxed versus tense in: FSP SCM 3km/h (*p* < 0.001); driver SCM 3km/h (*p* < 0.001)).

The analysis revealed a significant main effect of Δ*v* on the reflex amplitude (*p* < 0.0001). In all three muscles of the driver and FSP, a higher Δ*v* trended towards a greater reflex amplitude (Fig 3, left vs. right). The post-hoc tests showed that this trend reached significance in the SCM and in the TRA muscles of both the driver and the FSP in relaxed as well as in tense condition (Fig 3, compare 3 versus 6 km/h in: driver SCM relaxed (*p* < 0.001), driver SCM tense (*p* < 0.01), driver TRA relaxed (*p* < 0.0001), driver TRA tense (*p* < 0.01), FSP SCM relaxed (*p* < 0.0001), FSP SCM tense (*p* < 0.05), FSP TRA relaxed (*p* < 0.0001), FSP TRA tense (*p* < 0.0001)). Additionally, there was a significant difference between 3 km/h and 6 km/h in the driver’s PARA muscles on the relaxed condition (*p* < 0.001).

### Reflex delay

As for the reflex delay (Fig 4), the GLMM analysis (Table 4) revealed a significant interaction effect between the three variables muscle type, Δ*v*, and seating position (*p* < 0.05). Moreover, there was a significant interaction between muscle type and Δ*v* (*p* < 0.0001), and between muscle type and tension (*p* < 0.05).

There was no significant main effect of the seating position (*p* > 0.18) nor of Δ*v* (*p* > 0.81). There was a significant main effect of muscle pre-tension on the reflex delay (*p* < 0.01). Indeed, for the PARA and SCM muscles of both the driver and the FSP, pre-relaxed muscles trended towards shorter activation times than pre-tensed muscles, a trend that does not show up for the TRA muscles (Fig 4, top vs. bottom). It is this difference between the conditional effects that reflects the significant interaction between muscle type and tension. It should be noted, however, that the post-hoc tests did not reveal significance for this observed trend.

### Influence of the belt on the cervical muscle activity

The GLMM analyses neither revealed a significant main effect of the independent variable ‘belt’ on the reflex amplitude (*p* > 0.85, Fig 5, top) nor the reflex delay (*p* > 0.51, Fig 5, bottom).

### Motion of the head relative to the torso

The LMM analysis revealed a significant main effect of the seating position on the path length of the movement of the head relative to the torso (*p* < 0.05) as well as a significant interaction of seating position and muscle tension (*p* < 0.01). The post-hoc tests showed a significant difference (*p* < 0.01) on the ‘tense’ condition (Fig 6, left).

There was a significant main effect of the seating position on the maximum velocity of the head relative to the torso (*p* < 0.01). The post-hoc tests, though, revealed no significant differences between the individual factor levels (Fig 6, middle).

No significant main effect of the seating position on the maximum acceleration of the head relative to to the torso (Fig 6, right) could be found (*p* > 0.17).

### Comparison of effect sizes

When seating position, Δ*v*, and muscle tension are ranked according to the size of their effect on the neck muscle activation in terms of Cramer’s *ϕ*, we obtain the following picture: The lowest effect is produced by the seating position, as it has no significant effect at all (hence its effect size is technically zero), neither on the reflex delay nor on the reflex amplitude. Tension has a small effect on reflex delay (*ϕ* = 0.09) and a medium effect on reflex amplitude (*ϕ* = 0.15). The change in velocity Δ*v* has no significant effect on the reflex delay but a large one, in this comparison even the largest one, on the reflex amplitude (*ϕ* = 0.34).

## Discussion

The purpose of this study was to examine the muscular responses of driver and front seat passenger (FSP) during low-velocity left-frontal-oblique collisions, to gain further insights into the neuromuscular mechanisms potentially related to whiplash-associated disorders (WAD).

Systematic differences in the delay and/or the amplitude of the reflexes would have provided important insights into the neuromuscular mechanisms that underly the risk of WAD. However, due to the similarity of the driver’s and FSP’s muscle activity responses such conclusions are not possible.

An important question is why the reflex delays and amplitudes did not differ between the seating position of the driver and the FSP. One possible explanation is that the risk of WAD in frontal-oblique collisions is not different. The driver’s increased WAD risk has been found in rear-end collisions [19, 20], whereas it has not been verified that this risk difference is also present in every other collision direction. Alternatively, the high predictability of the collisions in the present experimental design might have reduced any differences in muscular responses between the driver and FSP position. As the drivers in this study faced the inevitability of the upcoming collision, they may have experienced the situation not being in a proactive role but rather taking a passive role like FSPs do.

In addition to the mechanical stimuli affecting all vehicle occupants, grasping the steering wheel provides further afferent information exclusively for the driver. Various mechanoreceptors in the palm of the hands and in the muscles of the upper limbs may be stimulated by a vibration or acceleration of the steering wheel towards the driver and thus affect the cervical muscle response [26]. Although this afferent information is likely to be available in an early stage of the mechanical sequence of the perturbation, it did not lead to a significantly earlier reflex onset of the driver (Fig 4). Therefore, the impact of the afferent information provided by the steering wheel, on the neck muscle activity onset seems to be relatively small.

In all of the four different experimental conditions, the TRA muscles trended towards a greater reflex amplitude in the drivers as compared to the FSPs (Fig 3). Due to the collision-induced approaching of the steering wheel to the driver, the extensor muscles of the elbow joints are likely to tense reflexively [44], because such a response would decelerate the forward acceleration of the upper body. The stiffened upper limbs may then, mediated by the shoulders, invoke a mechanical stimulus to the TRA muscles. In the relaxed as well as in the tense condition, the driver trends to make smaller movements, velocities, and accelerations than the FSP (Fig 6). This trend is even more apparent in the tense condition. Thus, when the occupants were asked to brace for the impact, it seems that, by stiffening the cervical spine, the steering wheel helped the drivers to reduce the movement of the head relative to the torso.

As for the influence of the belt, the orientation of the customary 3-point lap and shoulder belt is different in the driver and FSP position (Fig 2A). While in frontal, rear-end, and lateral collision directions the influence of the shoulder belt’s asymmetry is expected to be minor, oblique impact directions may interact with an asymmetrical shoulder belt orientation in an exacerbating manner, and this interaction may affect the driver and the FSP differently. Restraining one shoulder more effectively than the other, the asymmetry of the 3-point shoulder belt may represent a source of the higher risk of drivers to suffer from WAD compared to FSPs, if it exists in frontal-oblique collisions.

However, the comparison of the cervical muscle activity in the belted and unbelted conditions showed that the belt neither had a significant influence on the reflex amplitude nor on the reflex delay (Fig 5). As not even the fact of being belted or not led to a difference in the EMG data, the belt’s orientation is rather unlikely to provoke different cervical muscle activities. Thus, although we did not investigate the opposite shoulder belt orientation (top-left to bottom-right for FSP and top-right to bottom-left for driver), neither the belt nor its orientation seem to be relevant regarding a higher WAD risk of drivers. These results are in line with Kumar et al. 2006 [45] who did not find significant differences in the muscular responses of subjects using a symmetrical 5-point shoulder belt and subjects using an asymmetrical 3-point shoulder belt following eight different (straight and oblique) impact directions.

To summarize the above reasoning, our EMG results do not provide an explanation for a potential higher WAD risk of drivers compared to FSPs following frontal-oblique collisions. Future studies should compare the neck muscle activities of driver and FSP during rear-end collisions, especially since a larger backset (horizontal distance between head restraint and back of the head) in the driver’s position is expected to play a more important role in rear-end collisions than in other impact directions [13, 20, 46]. In addition, the effect of the steering wheel should be investigated more precisely as our results suggest an influence of the steering wheel on the neck muscle activity and the head-torso kinematics.

Concerning the effect of muscle pre-tension on the reflex response of the cervical muscles, we get the following results. In drivers as well as in FSPs, pre-tensioning increased the muscle activation onset by about 20 ms in the PARA and SCM muscles (except PARA of FSP in the 3 km/h condition), but barely affected the reflex delay of the TRA muscles (Fig 4). These results refute our hypothesis H2b, and differ from the results of Siegmund et al. (2003) [37] (aware vs. unaware) and Magnusson et al. (1999) [38] (expected vs. unexpected), who reported comparable muscle activation timings. However, our results may be explained as follows. Reflexive coactivation of muscles that work in an agonist-antagonist relation had been shown to result in a delayed reflex onset [47]. This delay may be caused by an interaction of conflicting commands such as the simultaneous activation and inhibition of the same muscle. Accordingly, pre-tensioning SCM and PARA muscles may cause a reciprocal inhibition of *α*-motor neurons, which might be responsible for the later onset timing compared to initially relaxed muscles. The results of the response delay times, however, may also have a purely methodological explanation. As the pre-tension causes a higher baseline activity prior to the perturbation onset, the reflex gain will increase not as steep as in initially relaxed muscles, which are characterized by a low baseline activity. The steeper the increase of the muscular activation, the earlier the reflex onset is detected.

As for the response amplitude, taking a tense body posture prior to the impact increased the reflex amplitude in drivers as well as in FSPs in all three examined muscles. Thus, in the tense condition the impact-induced muscle activity seems simply to be added to the pre-impact muscle activity, especially in the 3 km/h condition.

In summary, the pre-tensioning has a significant effect on the reflex amplitude and delay, but from there it cannot be concluded whether this effect is influential on the risk or severity of sustaining WAD.

Next, in all three investigated cervical muscles, drivers as well as FSPs reveal greater reflex amplitudes for higher Δ*v* (Fig 3), as hypothesized. This result can be associated with a graded response amplitude to stimulus intensity because of the need to control a larger perturbation [9].

The results of the reflex delay, however, are less consistent. The GLMM analysis did not reveal a significant main effect of Δ*v*, but a significant interaction between Δ*v* and muscle type, which corresponds to the different behavior of PARA muscles compared to SCM and TRA muscles, when Δ*v* is increased (Fig 4, left vs. right): The SCM and TRA muscles trend towards shorter reflex delays, while the PARA muscles remain constant (on the relaxed condition) or even increase their delay (on the tense condition). The latter finding may be explained as follows. The overall shorter reflex delay for SCM and TRA at 6 km/h indicates that the stimulus was detected by the central nervous system earlier in the 6 km/h condition than in the 3 km/h condition. The shorter time interval between initial car acceleration and occupant acceleration at higher Δ*v* is likely to explain this result [9] as in a higher change in velocity the neural threshold of the perturbation detecting sensors is probably exceeded earlier. However, in the PARA muscle the trend towards a constant (relaxed condition) or even shorter (tense condition) activity onset at the 3 km/h level compared to the 6 km/h level defies this explanation.

## Conclusion

We investigated the influence of change in velocity (Δ*v*), seating position, muscle pre-tension, and the belt on the muscular response amplitude and delay of neck muscles in frontal-oblique collisions. Due to the similar EMG results in the belted and unbelted condition (Fig 5), neither the belt nor its asymmetrical orientation had an influence on the cervical muscle response of the occupants. In summary, we did not find significant differences in the reflex amplitude and delay of the neck musculature of the driver compared to the front seat passenger. We, therefore, conclude that an increased risk of the driver sustaining WAD in frontal-oblique collisions, if it exists, cannot be due to differences in the reflexive response of the occupants.

## Ethics statement

The study was approved by the ethics committee of the Institute of Sports Science and Psychology of the University of Münster (2015-44-HW). All subjects gave written informed consent after having been thoroughly informed about the nature and course of the experiment.
